# Circulating microparticle concentrations across acute and chronic cardiovascular disease conditions

**DOI:** 10.14814/phy2.14534

**Published:** 2020-08-03

**Authors:** Rian Q. Landers‐Ramos, Odessa A. Addison, Brock Beamer, Leslie I. Katzel, Jacob B. Blumenthal, Shawn Robinson, James M. Hagberg, Steven J. Prior

**Affiliations:** ^1^ Department of Kinesiology Towson University Towson MD USA; ^2^ Division of Gerontology and Geriatric Medicine Department of Medicine University of Maryland School of Medicine Baltimore MD USA; ^3^ Baltimore Veterans Affairs Geriatric Research Education and Clinical Center Baltimore MD USA; ^4^ Department of Kinesiology University of Maryland College Park MD USA

**Keywords:** cardiovascular disease, coronary artery disease, endothelial microparticles, microparticles, NSTEMI

## Abstract

Concentrations of different circulating microparticles (MPs) may have clinical and physiological relevance to cardiovascular disease pathologies.

**Purpose:**

To quantify plasma concentrations of CD31+/CD42b−, CD62E+, and CD34+ MPs across healthy individuals and those with coronary artery disease (CAD) or acute cardiovascular events (non‐ST elevation myocardial infarction (NSTEMI)). Fasted blood was obtained from CAD patients (*n* = 10), NSTEMI patients (*n* = 13), and healthy older men (*n* = 15) 60–75 years old.

**Methods:**

CD31+/CD42b−, CD62E+, and CD34+ MPs were isolated from plasma and quantified using flow cytometry. Relationships between MP subtypes, fasting blood lipids, blood glucose, blood pressure, body mass index, and total number of medications were assessed.

**Results:**

Concentrations of CD31+/CD42b− MPs were significantly lower in CAD and NSTEMI subjects compared with healthy individuals (*p* = .02 and .003, respectively). No differences between groups were found for CD62E+ or CD34+ MPs (*p* > .05 for both). Surprisingly, among all variables assessed, only CD62E+ MP concentrations were positively correlated with triglyceride levels (*p* = .012) and inversely correlated with SBP (*p* = .03).

**Conclusions:**

Our findings provide support for the use of different MP subtypes, specifically CD31+/CD42b− MPs, as a potential biomarker of cardiovascular disease. Importantly, results from this study should be looked at in adjunct to previous MP work in CVD conditions as a way of highlighting the complex interactions of variables such as comorbid conditions and medications on MP concentrations.

## Graphical abstract

In contrast to CD62E+ and CD34+ MPs, CD31+/CD42b− MPs were significantly lower in CAD and NSTEMI patients versus healthy older adults. These findings suggest CD31+/CD42b− MP concentration as a potential biomarker of cardiovascular disease and highlight the need for a better understanding of comorbidities and medications on MP concentrations.

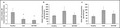

**Abbreviations**
ANOVA: analysis of variance
BMI: body mass index
CAD: coronary artery disease
CFP: cell‐free plasma
CV: cardiovascular
CVD: cardiovascular disease
DBP: diastolic blood pressure
EDTA: ethylenediamine tetraacetic acid
FITC: fluorescein isothiocyanate
HDL‐C: high‐density lipoprotein cholesterol
LDL‐C: low‐density lipoprotein cholesterol
MP: microparticle
NSTEMI: non‐ST elevation myocardial infarction
PE: phycoerythrin
SBP: systolic blood pressure
*SEM*: standard error of the mean
Shh: sonic hedgehog
TG: triglyceride
UM‐SOM: University of Maryland School of Medicine
VA: Veterans Affairs

## 1 INTRODUCTION

Cardiovascular disease (CVD) is expected to affect over 40% of adults in the United States by 2030 (Heidenreich et al., 2011). With only half of CV events explained by traditional CVD risk factors (Mora, Cook, Buring, Ridker, & Lee, 2007), biomarkers that extend beyond traditional risk factors may be critical to explain CVD activity (Koganti et al., 2017). Cellular endothelial microparticles (MPs) have emerged as novel biomarkers and may serve in a clinical capacity to identify CVD burden (Markiewicz, Richard, Marks, & Ludwicka‐Bradley, 2013). Endothelial MPs are small particles (~0.1‐1 µm) shed from the vascular endothelium and circulating cells in response to inflammation and apoptosis (Chironi et al., 2009; Horstman, 2004). MPs contain nuclear materials that play a role in cellular signaling (Dignat‐George & Boulanger, 2011). Once released, the MP contents can promote further damage to the target tissue through endothelial activation or may work in a compensatory manner to promote endothelial differentiation and survival (Boulanger, Loyer, Rautou, & Amabile, 2017; Dignat‐George & Boulanger, 2011; Paudel, Panth, & Kim, 2016; Puddu, Puddu, Cravero, Muscari, & Muscari, 2010). Thus, the concentrations of different MPs in plasma may have clinical and physiological relevance to different CVD pathologies and severity.

MP surface markers may provide some indication regarding the mechanism of release. CD31+/CD42b− MPs express endothelial surface markers of nonplatelet origin and are released by apoptotic cells (Chironi et al., 2009; Horstman, 2004). Elevated circulating concentrations of these MPs have been found with hypertension, acute coronary syndrome (Preston et al., 2003), and type 2 diabetes (Landers‐Ramos et al., 2018) and have also been linked to functional CV outcomes including impaired arterial elasticity in apparently healthy subjects (Wang et al., 2007). CD62E+ MPs originate from the endothelium and are shed in response to cellular activation (Chironi et al., 2009; Horstman, 2004). Individuals with coronary artery disease (CAD) exhibit significantly greater mean percentage of CD62E+ MPs compared to healthy adults (Hu, Zhang, Zhang, & Xiu, 2014). CD34+ MPs released from endothelial cells or hematopoietic progenitor cells may also serve as potential clinical biomarkers. Some studies suggest that CD34+ MPs may exert pro‐angiogenic properties of their parent cells or work in a compensatory manner to offset the effects of other MPs (Lansford et al., 2016). We previously reported that CD34+ MPs are elevated in older adults compared to younger adults (Landers‐Ramos et al., 2018) and elevated CD34+ MPs have been found in individuals with a recent acute myocardial infarction compared to healthy adults and those with stable angina (Stepień et al., 2012). However, the concentration of CD34+ MPs in relation to other MP subtypes traditionally representing CV burden is not well understood.

Collectively, endothelial dysfunction and low‐grade inflammation associated with chronic CVDs such as CAD may result in elevations in various MP subtypes which could be an indicative of the type of endothelial damage and serve as important biomarkers for these conditions (Markiewicz et al., 2013). Furthermore, an acute cardiac event may result in greater elevations of certain MPs, perhaps indicative of a need for enhanced cellular signaling (Stepień et al., 2012). Although differences in the concentrations of MP subtypes have been identified in several disease cases including CVDs, a comparison of multiple MP subtypes among healthy individuals, those diagnosed with CVD, and those recently suffering from an acute CV event has not been documented. The purpose of this study was to compare plasma concentrations of CD31+/CD42b− MPs, CD62E+ MPs, and CD34+ MPs across CAD, acute CV events (non‐ST elevation myocardial infarction (NSTEMI)), and healthy controls. We hypothesized that each MP subtype would be elevated in each CVD population compared with healthy controls and that MP concentrations would be highest in the NSTEMI patients.

## 2 MATERIALS AND METHODS

### 2.1 Ethical standards

Study procedure was approved by the University of Maryland School of Medicine (UM‐SOM) Institutional Review Boards (HP00057124) and were carried out in accordance with the Declaration of Helsinki and its later amendments or comparable ethical standards. All subjects provided informed verbal and written consent.

### 2.2 Screening and standard assessments

Subjects were recruited from the greater Baltimore and Washington DC area. All subjects were men 60–75 years old and were sedentary, completing ≤20 min of exercise on ≤2 days per week. Exclusion criteria for the healthy group were as follows: smoking, known CVD, type 1 or type 2 diabetes, chronic obstructive pulmonary disease, current treatment for active cancer, systolic blood pressure ≥ 130 mmHg, diastolic blood pressure ≥ 90 mmHg, serum total cholesterol ≥ 200 mg/dl, low‐density lipoprotein cholesterol (LDL‐C) ≥130 mg/dl, high‐density lipoprotein cholesterol (HDL‐C) ≤35 mg/dl, or fasting glucose ≥100 mg/dl. NSTEMI patients had a documented NSTEMI and were tested within 24–72 hr of presentation to the Emergency Room. Potential NSTEMI patients were recruited from those admitted to the Baltimore Veterans Affairs (VA) Medical Center. The CAD subjects were age‐matched to the NSTEMI group and recruited from the same medical center with a medically documented history of or current symptomatic CAD. Participants were not excluded based on race, ethnicity, or medications, due to their necessity in these populations. Subjects from the CVD groups were not excluded due to other chronic conditions including high blood pressure, hyperlipidemia, or hyperglycemia, and these conditions and accompanying medications were documented.

### 2.3 Fasted blood draw

Blood draws were performed the morning after an overnight (12‐hr) fast. Blood samples from NSTEMI patients were obtained between 24 and 72 hr after presentation to the Emergency Room. Blood was collected in tubes containing 15% potassium ethylenediamine tetraacetic acid (EDTA) as described previously (Jenkins et al., 2011; Landers‐Ramos et al., 2018). After centrifugation, plasma was isolated for measurement of lipoprotein‐lipid levels. Plasma for MP measures was isolated and stored at −80°C until future analysis.

### 2.4 Blood sample analyses

Plasma glucose levels were measured in duplicate using the glucose oxidase method (2,300 STAT Plus. YSI. Yellow Springs). Plasma triglyceride (TG) and cholesterol were analyzed by enzymatic methods (Hitachi mofel‐917 analyzer). HDL‐C was measured in the supernatant after precipitation with dextran sulfate, and LDL‐C was calculated using the Friedewald equation (Friedewald, Levy, & Fredrickson, 1972).

### 2.5 Plasma microparticle quantification and analysis

MP quantification and analysis were performed as previously described (Jenkins et al., 2011; Landers‐Ramos et al., 2018) with minor modifications. Briefly, plasma samples were thawed, and platelet‐poor plasma was obtained through centrifugation. Platelet‐poor plasma was then further centrifuged to obtain cell‐free plasma (CFP), which was used for MP analyses. Fifty‐microliters of CFP was incubated with anti‐CD31‐phycoerythrin (PE) and anti‐CD42b‐fluorescein isothiocyanate (FITC), anti‐CD62E‐PE, or anti‐CD34‐FITC (BD Biosciences) for 30 min. Samples were then fixed with 2% paraformaldehyde and diluted with filtered PBS before flow cytometric analysis.

MP quantification was performed using an LSR II flow cytometer with a lower detection limit of 0.5 µm. Samples were each analyzed for 3 min at a medium flow rate. Countbright Absolute Counting Beads (ThermoFisher Scientific) were added to each sample immediately before quantification and ultra‐pure water was run for 1 min in between each sample. MPs were defined as CD31+/CD42b−, CD62E+, or CD34+ events between 0.5 and 1.0 µm, excluding exosomes and other small microvesicles from analyses (Gould & Raposo, 2013). A logarithmic scale was used for forward scatter signal, side scatter signal, and each fluorescent channel and 0.9 µm standard precision NIST traceable polystyrene particle beads (Polysciences, Inc) were used for size calibration and forward scatter signal. Proper color and size controls were used to distinguish true events from background noise. Controls and gating strategies were performed as previously reported (Landers‐Ramos et al., 2018). MPs were quantified using Winlist 6.0 (Verity Software). The concentration of MPs was calculated using Countbright Absolute Counting Beads (ThermoFisher Scientific).

### 2.6 Statistical analysis

Statistical analyses were completed using IBM SPSS version 21 (Armonk, NY). Assumptions of homoscedasticity and normality were evaluated for all measures. MP data were log‐transformed for statistical analyses due to a non‐normal distribution of residuals in raw data. Differences among groups were compared using one‐way ANOVAs and analyses of covariance were used to control for factors significantly correlated with MP subtype. Pearson correlation coefficients were calculated for log values of plasma MP and values of blood lipids, blood pressure, body mass index (BMI), glucose, and total number of medications. Factors that significantly correlated with MPs were further assessed in a multivariable regression. Statistical significance was accepted at *p* ≤ .05. MP data are expressed as means ± standard error of the mean (*SEM*).

## 3 RESULTS

### 3.1 Subject characteristics

Subject characteristics can be found in Table 1. NSTEMI subjects were significantly older than the healthy subjects (*p* = .016). The CAD subjects had significantly higher TG compared to both the healthy subjects (*p* = .021) and the NSTEMI patients (*p* = .05). The number of participants with type 2 diabetes mellitus was significantly higher in both the CAD and the NSTEMI groups compared to the healthy controls (*p* = .01 and *p* = .021, respectively), although fasting glucose concentrations were not different. There was a significant difference in the total number of medications taken between groups with healthy participants taking significantly fewer medications than the CAD subjects (*p* = .01). A list of all prescribed medications can be found in Table 2.

**TABLE 1:** Subject characteristics

	Healthy (*n* = 15)	CAD (*n* = 10)	NSTEMI (*n* = 13)
Age	63 ± 2	69 ± 2.6	71 ± 2.6^*^
Body mass index (kg/m^2^)	29 ± 0.9	31 ± 2	26 ± 1.3
Cholesterol (mg/dl)	188 ± 8.8	171 ± 10.1	169 ± 18.2
HDL cholesterol (mg/dl)	51 ± 4.2	43 ± 4.3	45 ± 2.7
LDL cholesterol (mg/dl)	120 ± 7	90 ± 10.9	102 ± 15.4
Triglyceride (mg/dl)	87 ± 12.9	189 ± 44.2^*^	118 ± 23.6^#^
Glucose (mg/dl)	96 ± 2.2	103 ± 3	113 ± 8.4
Systolic blood pressure (mm Hg)	121 ± 4.8	133 ± 4.7	131 ± 4.9
Diastolic blood pressure (mm Hg)	75 ± 1.6	74 ± 2.7	71 ± 2.8
Mean arterial pressure (mm Hg)	90 ± 1.9	94 ± 2.7	91 ± 2.8

Abbreviations: HDL, high‐density lipoprotein; LDL, low‐density lipoprotein.

* *p* ≤ .05 compared to healthy.

^#^ *p* = .05 compared to CAD.

**TABLE 2:** Medications

		Healthy	CAD		NSTEMI	
# Subjects taking Medications, *n* (%)						
Statin		5 (33)	7 (70)		5 (38)	
Fish Oil		3 (20)	3 (30)		0 (0)	
Orlistat		1 (7)	0 (0)		0 (0)	
Other lipid lowering		0 (0)	0 (0)		0 (0)	
Aspirin alone		3 (20)	5 (50)		8 (62)	
Antiplatelet alone		0 (0)	2 (20)		0 (0)	
Dual Antiplatelet		0 (0)	3 (30)		1 (8)	
No antiplatelet		11 (73)	1 (10)		4 (31)	
Anticoagulant		0 (0)	1 (10)		1 (8)	
ACEi/ARB		4 (27)	4 (40)		5 (38)	
Beta‐Blocker		2 (13)	5 (50)		5 (38)	
Calcium channel blocker		4 (27)	2 (20)		3 (23)	
Other Vasodilator^*^, ^a^		0 (0)	3 (30)		2 (15)	
Sotalol + Digoxin		0 (0)	0 (0)		1 (8)	
Diuretic		3 (20)	5 (50)		5 (38)	
No CV med at all		6 (40)	0 (0)		1 (8)	
Glucose lowering		0 (0)	4 (40)		3 (23)	
Neurologic/Psychiatric		1 (7)	5 (50)		1 (8)	
Vitamin/Mineral/Supplement		5 (33)	3 (30)		2 (15)	
GI Medication^#^, ^b^		2 (13)	2 (20)		2 (15)	
NSAID Analgesic^#^, ^b^		1 (7)	3 (30)		0 (0)	
Allopurinol		0 (0)	3 (30)		0 (0)	
Levothyroxine		1 (7)	1 (10)		1 (8)	
Anti‐Androgen		1 (7)	3 (30)		1 (8)	
Inhaled/ Nasal corticosteroid^#^, ^b^		0 (0)	1 (10)		4 (31)	
Montelukast		1 (7)	0 (0)		0 (0)	
Bronchodilator^#^, ^b^		2 (13)	5 (50)		1 (8)	
Antihistamine^#^, ^b^	1 (7)		2 (20)	2 (15)	
Antibiotic^#^, ^b^	1 (7)		0 (0)		0 (0)	
Number of total medications (Mean ± *SEM*)	4 ± 1.1		9.4 ± 1.3^*^		5.7 ± 1.1	

Abbreviations: ACEi, angiotensin‐converting enzyme inhibitor; ARB, angiotensin II receptor blocker; CV, cardiovascular; GI, gastrointestinal; NSAID, nonsteroidal anti‐inflammatory drug.

^a^ Alpha‐1‐adrenergic blocker; Hydralazine; Long‐acting nitrate.

^b^ Some of these were prescribed for use as needed. Time of most recent dose is not available.

* *p* ≤ .05 compared to healthy.

### 3.2 Microparticle concentrations

#### 3.2.1 **CD31+/CD42b**−** Microparticles**

**FIGURE 1:**
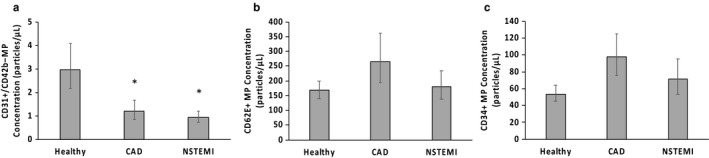
Comparison of CD31+/CD42b− (a), CD62E+ (b), and CD34+ (c) microparticle (MP) concentrations in healthy adults (*n* = 15), coronary artery disease (CAD; *n* = 10), and non‐ST elevation myocardial infarction (NSTEMI; *n* = 13). CD62E+ MPs are depicted after covarying for triglycerides and systolic blood pressure. Raw data are presented but were log‐transformed for analyses. Data represent mean ± *SEM*. ^* ^Represent significantly different from healthy adults (*p* < .05)

CD31+/CD42b− MPs were 60% and 69% lower in both the CAD (1.19 ± 0.49 particles/µl) and the NSTEMI patients (0.94 ± 0.26 particles/µl), respectively, compared with the healthy group (2.98 ± 1.1 particles/µl; *p* = .02 and *p* = .003, respectively). No statistically significant differences were found between the CAD and NSTEMI groups (*p* = .57; Figure 1a).

#### 3.2.2 CD62E+ Microparticles

There was no main effect of group for CD62E+ MP concentrations initially (*p* = .38) or when controlling for TG and systolic blood pressure (SBP; *p* = .40). CD62E+ MP concentrations appeared numerically higher in the CAD group (265.6 ± 96.1 particles/µl) compared with the healthy subjects (167.6 ± 32.1 particles/µl), but this did not reach statistical significance (*p* = .19; Figure 1b); the healthy subjects and the NSTEMI patients (179.2 ± 54.1 particles/µl) exhibited similar concentrations (*p* = .76).

#### 3.2.3 CD34+ Microparticles

There was no group main effect for CD34+ MP concentrations (*p* = .125), but when individual groups were compared the CAD group had 82% more CD34+ MPs than the healthy group (*p* = .05). Concentrations of CD34+ MPs in the NSTEMI group (71.2 ± 24.4 particles/µl) were between those in the healthy (53.5 ± 10.5 particles/µl) and CAD (97.4 ± 27.7 particles/µl) groups but were not statistically different from either of those groups (*p* = .20 and .39, respectively) (Figure 1c).

### 3.3 Relationships among MP concentrations and CVD risk factors

**FIGURE 2:**
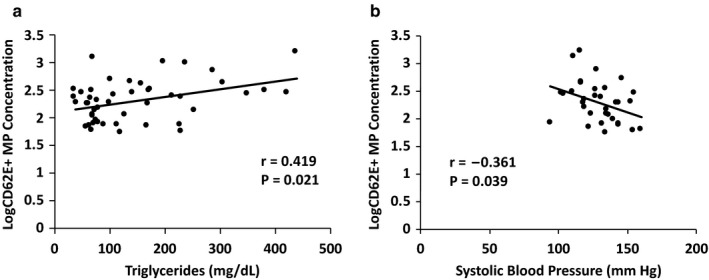
Correlations between CD62E+ microparticles and triglycerides (a) or systolic blood pressure (b). Log‐transformed MP data are presented

No significant correlations were observed between CD31+/CD42b− MP and the CD34+ concentrations versus any subject characteristics. CD62E+ MP concentrations were directly correlated with plasma triglyceride concentrations (*r* = .419; *p* = .012) and inversely correlated with SBP (*r* = −.361; *p* = .03). When entered into a regression analysis, both triglycerides and SBP remained statistically significant predictors of CD62E+ MP concentrations (Figure 2a and b).

## 4 DISCUSSION AND CONCLUSIONS

In this study we demonstrate substantially lower concentrations of CD31+/CD42b− MPs in CAD and NSTEMI subjects compared with healthy individuals, lending support for the use of CD31+/CD42b− MPs as potential biomarkers of CVD. We also found that CD62E+ MP concentrations were directly associated with triglyceride levels while they were inversely associated with SBP in all subjects regardless of group. Surprisingly, no significant differences in CD34+ or CD62E+ MP concentration were found across groups even after controlling for covariates.

### 4.1 CD31+/CD42b− MPs

Surprisingly, we found that both CAD and NSTEMI patients exhibited lower CD31+/CD42b− MP concentrations compared with healthy participants. These findings are in conflict with our original hypothesis and previous literature demonstrating elevations in CD31+/CD42b− MP concentrations in individuals with acute coronary syndrome (Bernal‐Mizrachi et al., 2003; Preston et al., 2003) and in those with an acute MI (Mallat et al., 2000) compared to healthy age‐matched adults. Interestingly, Bernal‐Mizrachi et al. found that CD31+ MPs were substantially higher in patients suffering from their first MI as compared with patients with recurring MI (Bernal‐Mizrachi et al., 2003), and that MP number correlated with the number of lesions measured using coronary angiography (Bernal‐Mizrachi et al., 2003). In the current study, the degree of myocardial damage from the present NSTEMI or any previous cardiac events was not assessed. It is possible that repeated insults with recurring acute coronary events in this group and potentially chronic CAD would lead to “endothelial exhaustion” and a reduction in CD31+/CD42b− MPs (Bernal‐Mizrachi et al., 2003). From a physiological perspective, a reduction in MPs carrying nuclear materials may contribute to worsened endothelial function due to reduced signaling from MP contents and potentially a low turnover of healthy endothelial cells. Additionally, some MP subtypes are known to bind to monocytes which promotes an inflammatory response via enhanced transendothelial migration and reduces the concentration of free MPs (Horstman, 2004; Jy, 2004). These theories may explain our observed low concentration of CD31+/CD42b− MPs in CAD and NSTEMI patients compared to our healthy controls. However, future empirical studies are needed to determine this.

### 4.2 CD62E+ MPs

In our study, we report no statistically significant differences in CD62E+ MP concentrations across groups. We are aware of at least one previous report of elevated CD62E+ MPs in CAD patients compared to healthy individuals (Hu et al., 2014) and numerically, our findings are similar. Hu et al. used a slightly larger samples size which may explain the statistical differences between this and the current study. Previous work has also reported no differences in MP levels between CVD populations and healthy controls (Giarretta et al., 2018). Interestingly, Giaretta et al. found that endothelial MPs from some CVD patients carried more sonic hedgehog (Shh) protein, a key regulator of angiogenesis, compared to healthy controls (Giarretta et al., 2018). Differences in contents of MPs from CVD groups compared to healthy controls clearly could play a physiological role in the regulation of angiogenesis and/or endothelial function, despite no differences in MP concentration. A comprehensive analysis of MP contents including proteins, mRNAs, and microRNAs from various CVD conditions is needed to fully understand the physiological role of MPs in CVD conditions.

It is worth acknowledging the high number of medications taken by all of the patient populations in this study. It is possible that a combination of medications prescribed to these subjects may have influenced our findings. For example, in addition to their prescribed daily medications, NSTEMI patients were provided with medications to treat the acute MI upon arrival to the emergency room and before cardiac catheterization procedures took place. There have been previous reports of clinically relevant concentrations of statins increasing MP concentrations using a cell culture model (Diamant et al., 2008), while other studies have found that statins and calcium channel blockers reduce MP concentrations (Nomura, 2008; Tramontano et al., 2004). Our study is strengthened by the clinical relevance of not just the patient populations, but in scenarios commonplace for many older clinical populations (i.e., multiple prescribed medications). No studies to our knowledge have investigated interactions between multiple medications and their role in potentially altering different MP concentrations. Thus, it is difficult to determine whether combination of medications or medications given acutely to the NSTEMI patients may have affected our study outcomes. More research into drug interactions in disease populations is needed to better determine the use of MPs as true clinical biomarkers.

CD62E+ MPs were the only MP subtype related to any documented risk factors. The positive correlation found between CD62E+ MPs and TG in this study supports previous reports of elevated triglycerides and endothelial impairment (Lundman et al., 2003). Interestingly, we also observed an inverse association between CD62E+ MPs and SBP. While this appears to contradict reports of elevated MPs in hypertensive individuals (Sansone et al., 2018), it is possible that medications used to control blood pressures in the CAD and NSTEMI groups attenuate MP release. This provides further support for the need to better understand the role of medications on MP concentrations.

We have recently shown that elevations in CD31+/CD42b− and CD62E+ MPs are driven by type 2 diabetes (Landers‐Ramos et al., 2018). In the current study, although type 2 diabetes diagnoses were higher in both of the CVD groups compared with the healthy group, fasting glucose levels were similar, which differ from our previous report (Landers‐Ramos et al., 2018). Thus, the medically controlled blood glucose levels in the current study may explain why these MP concentrations are not elevated in the patient populations having a greater prevalence of type 2 diabetes compared to healthy controls. Multiple comorbidities are common in older adults (Blackwell, Lucas, & Clarke, 2014). While more concrete conclusions regarding mechanisms are possible by isolating single conditions, this study is strengthened by the inclusion of subjects who are representative of older adults with CVD. Importantly, since the goal of many researchers investigating MPs is for their use as clinical biomarkers, inclusion of subjects who represent the complexities that CVD presents (i.e., multiple comorbidities and medications) is critical.

### 4.3 CD34+ MPs

CD34+ MPs are a less‐commonly studied MP population and the exact function and/or stimulus‐causing alterations in circulating concentrations is still not completely understood. In an atherogenic environment, circulating CD34+ cells exhibit characteristics of foam cells which can further exacerbate the development of atherosclerosis (Daub et al., 2010) and potentially result in the release of CD34+ MPs from these foam cells or from mature activated endothelial cells. In the current study, we report no significant differences in CD34+ MP concentrations across groups, although numerically, CD34+ MPs were higher in CAD patients compared to healthy individuals. Given these findings, it is possible that a larger sample size would reveal statistical differences between these populations. Mechanistic studies to determine the role of an atherogenic environment on CD34+ MP concentrations (or vice versa) are needed to fully understand the potential role of CD34+ MPs in chronic and acute CVD conditions. We have previously reported elevations in CD34+ MPs in older compared with younger adults (Landers‐Ramos et al., 2018). It is possible that CD34+ MPs better serve as an indicator of the aging endothelium as opposed to CVD progression.

There have been few studies that demonstrate associations between MP subtypes and vascular function (Babbitt et al., 2013; Wang et al., 2007). However, the effectiveness of the use of MPs as clinical biomarkers for certain disease conditions is dependent on a demonstrated relationship between MP concentrations and functional outcomes in future work. Previous reports demonstrate the ability to detect significant differences in MP subtypes using similar or smaller sample sizes as used in this study (Jenkins et al., 2011; Landers‐Ramos et al., 2018; Lansford et al., 2016). However, we were limited in our ability to perform subanalyses within each group according to disease severity and comorbidities and still have enough statistical power to detect meaningful differences. All subjects in the current study were men due to the greater percentage of male patients seen at the VA hospital in which they were recruited. Future studies including women are needed to determine whether sex differences exist. Finally, it should be mentioned that despite having diagnosed CAD or NSTEMI, the general risk factor profile for these patient populations is low. This along with the various medications may, in part, explain why our findings differ from previous findings and our original hypothesis.

In conclusion, our findings provide support for the use of different MP subtypes as potential biomarkers to be used in the identification of CVD. Importantly, results from this study should serve as an accessory to previous MP work in CVD conditions as a way of highlighting the complex interactions of variables such as comorbid conditions and medications on MP concentrations.

## DISCLOSURES

The authors declare that they have nothing to disclose and no conflict of interest to report. All the authors have read and agree to the publication of this manuscript as submitted and this paper is not currently under review elsewhere.

## AUTHORS' CONTRIBUTIONS

RQL and SJP conceived and designed the research project; RQL, BB, LIK, JBB, and SJP collected the data and performed the experiments; RQ, OAA, and SJP analyzed the data; RQL, OA, and SJP interpreted the results; RQL and BB prepared the figures; RQL drafted the manuscript; RQL, OA, BB, LIK, JBB, SR, JMH, and SJP edited, revised, and approved the final version of the manuscript.
